# Use of GoPro point-of-view camera in intubation simulation—A randomized controlled trial

**DOI:** 10.1371/journal.pone.0243217

**Published:** 2020-12-01

**Authors:** Wenjun Koh, Deborah Khoo, Ling Te Terry Pan, Lyn Li Lean, May-Han Loh, Tze Yuh Vanessa Chua, Lian Kah Ti

**Affiliations:** 1 Department of Anaesthesia, Yong Loo Lin School of Medicine, National University of Singapore, Singapore, Singapore; 2 Department of Anaesthesia, National University Health System, Singapore, Singapore; University of Alberta, CANADA

## Abstract

**Introduction:**

Teaching endotracheal intubation is uniquely challenging due to its technical, high-stakes, and highly time-sensitive nature. The GoPro is a small, lightweight, high-resolution action camera with a wide-angle field of view that can encompass both the airway as well as the procedurist’s hands and positioning technique when worn with a head mount. We aimed to evaluate its effectiveness in improving intubation teaching for novice learners in a simulated setting, via a two-arm, parallel group, randomized controlled superiority trial with 1:1 allocation ratio.

**Methods:**

We recruited Year 4 medical students at the start of their compulsory 2-week Anesthesia posting. Participants underwent a standardized intubation curriculum and a formative assessment, then randomized to receive GoPro or non-GoPro led feedback. After a span of three months, participants were re-assessed in a summative assessment by blinded accessors. Participants were also surveyed on their learning experience for a qualitative thematic perspective. The primary outcomes were successful intubation and successful first-pass intubation.

**Results:**

Seventy-one participants were recruited with no dropouts, and all were included in the analysis. 36 participants received GoPro led feedback, and 35 participants received non-GoPro led feedback. All participants successfully intubated the manikin. No statistically significant differences were found between the GoPro group and the non-GoPro group at summative assessment (85.3% vs 90.0%, p = 0.572). Almost all participants surveyed found the GoPro effective for their learning (98.5%). Common themes in the qualitative analysis were: the ability for an improved assessment, greater identification of small details that would otherwise be missed, and usefulness of the unique point-of-view footage in improving understanding.

**Conclusions:**

The GoPro is a promising tool for simulation-based intubation teaching. There are considerations in its implementation to maximize the learning experience and yield from GoPro led feedback and training.

## Introduction

Teaching endotracheal intubation is uniquely challenging due to its technical, high-stakes, and highly time-sensitive nature. Tutor and student often do not share identical views of the airway and surrounding equipment, which makes it difficult for tutors to identify issues and suggest improvements [1], and limit the effectiveness of simulated training sessions. In the COVID-19 era, intubating in full personal protective equipment (PPE) is an additional obstacle, and there is a pressing need for healthcare workers (HCWs) to be trained in safe airway management of a confirmed or suspected COVID-19 patient [2].

These issues can be solved with a point-of-view camera system. A recent case series found that point-of-view cameras, the GoPro (GoPro, Inc, San Mateo, CA), and Google Glass^™^ (Google, Mountain View, California), were useful in teaching endotracheal intubation [3]. The value of a point-of-view camera in teaching intubation was previously recognised more than two decades ago with the commercial release of the Airway Cam^™^ (Airway Cam Technologies, Wayne, PA, USA), a head-mounted camera system worn next to the proceduralist’s dominant eye [4]. However, its widespread adoption was hindered by its cumbersome nature, coupled with its narrow field of view and interference with the proceduralist’s line-of-sight [5].

The GoPro is a small, lightweight, high-resolution action camera once mainly used by hobbyists in action sports, but has increasingly been utilised for a range of clinical education settings [6–9]. When worn with a head mount, it has a 149-degree wide-angle field of view that can capture the majority of visual information available to the wearer. Importantly, unlike the Google Glass, it is widely available, does not involve placement in front of the eye, offers a view encompassing both the airway as well as the procedurist’s hands and positioning technique, and is suitable for spectacle users.

This study aims to evaluate the effectiveness of a GoPro camera in improving intubation teaching in a manikin, as well as intubation skill retention. Specifically, the study focuses on intubation teaching at a preliminary level, on medical students prior to their exposure to real-world intubations.

## Materials and methods

We conducted a two-arm, parallel group, randomized controlled superiority trial with 1:1 allocation ratio in undergraduate medical students learning endotracheal intubation. The trial protocol was approved by the Institutional Review Board from National University of Singapore (NUS IRB B-15-274) and written informed consent was obtained as required. The study took place at National University Hospital, Singapore, a tertiary university hospital with 1200 beds and affiliated with Yong Loo Lin School of Medicine. Study design adhered to the applicable CONSORT guidelines.

### Participants

We recruited Year 4 medical students from Yong Loo Lin School of Medicine at the start of their compulsory 2-week Anesthesia posting, where students are formally taught endotracheal intubation as part of their curriculum. No exclusion criteria were specified, although students were allowed to opt out of the study. Randomization was performed via a computer-generated random number sequence.

As part of the school’s curriculum, participants were first required to learn endotracheal intubation via a standardized teaching session at the start of their anesthesia posting. This involved a 10-minute video-recording showing a standardized technique for endotracheal intubation. This was followed by small-group teachings with school tutors, and finally a self-directed practice session with the school’s intubation trainer manikins, for a total teaching time of approximately 3 hours. Our study was conducted immediately after this standardized teaching session, as well as 3 months after.

### Formative assessment

Participants were split into intervention and control groups to undergo a baseline formative intubation assessment. Both groups performed the formative assessment with a head-mounted GoPro Hero4 camera. Intubation was performed on a Laerdal^®^ Airway Management Trainer (Laerdal Medical AS, Stavanger, Norway) to simulate a normal airway. A C-MAC^®^ video laryngoscope (Karl Storz, Tuttlingen, Germany) with a Macintosh-typed blade was used for its ability to allow tutors to review the grade of intubation for purpose of feedback and assessment, as well as its capability for recording. Participants were instructed to use it as a regular direct laryngoscope with a Macintosh blade, without the assistance of the real-time C-MAC^®^ monitor, and perform direct laryngoscopy for intubation. Standardized intubation equipment was prepared for each participant in a tray, and reset between participants.

Each intubation attempt was video-recorded using another camera with a vantage viewpoint, and these attempts were observed by an independent assessor who was randomly assigned and blinded to the randomisation to avoid assessor bias. The assessor analyzed the video playback and assessed participants based on a standardized checklist. This was then used to determine primary, secondary and exploratory outcome data for the formative assessment. The assessor and tutors were qualified anaesthetists with at least 5 years of experience each, and have all taught airway modules for the affiliated medical school prior to this study.

### Debrief and feedback

After completion of the entire intubation process, a tutor provided one-to-one feedback to the participant based on their observations of their formative assessment attempt. The focus was on improving intubation technique and intubation success rate. Tutors in the GoPro group had access to both GoPro and C-MAC^®^ video laryngoscope footage, while tutors in the control group only had access to the C-MAC^®^ video laryngoscope footage. Each feedback session was allocated approximately 30 minutes.

### Summative assessment at 3 months

Participants were asked to return 3 months later for a summative assessment. The summative assessment was conducted with the same methodology as the formative assessment session, with both groups wearing the GoPro. All sessions were subsequently assessed as before by the blinded independent assessor.

Additionally, as an exploratory analysis to simulate a difficult airway, participants were asked to intubate a Difficult Airway Management Simulator MW-13 (Kyoto Kagaku, Kyoto, Japan).

At the end of the summative assessment, both groups of participants were provided with another one-to-one, GoPro led feedback of their intubation attempt. They were then provided with a 4-question survey and a feedback form regarding the use of GoPro in intubation teaching. Any other comments communicated verbally to the study team members were noted down ad verbatim.

### Outcomes and covariates

The primary outcomes were successful intubation, as well as successful first-pass intubation (defined as successful endotracheal intubation during a single, initial intubation attempt). Secondary outcomes were duration of time needed for successful intubation ("Time to Intubation"), intubation "Technique score", and intubation "Slickness score".

Time to Intubation was defined as the duration of time from removal of facemask (taken as the beginning of apnea) to removal of laryngoscope from mouth. Timing was done using the vantage viewpoint camera. For exploratory analysis, Time to Intubation was further subdivided into Laryngoscopy time (defined as duration of time from removal of facemask to picking up of endotracheal tube) and Tube placement time (defined as the duration of time from picking up of endotracheal tube to removal of laryngoscope from mouth).

The intubation "Technique score" was a 24-point intubation technique grading checklist created to assess the participants’ intubation technique holistically, and was based on the standardized teaching session all participants underwent (S1 Appendix). Points were given for intubation preparation technique ("Preparation score", 8 points), endotracheal tube placement technique ("Placement score", 9 points), post-intubation actions ("Post-intubation score", 6 points), as well as an overall score for awareness of oxygen saturation ("Awareness score", 1 point). It was successfully used in the pilot study described below. Lastly, as complementary subjective criteria are commonly used in intubation teaching studies [10, 11], assessors were asked to grade participants with an overall intubation "Slickness score" (out of 100 points), defined as how practised and confident the participant appeared while intubating. These scores were graded with the aid of the vantage viewpoint camera.

Other exploratory variables included laryngoscopy view, which was evaluated using the Cormack-Lehane Grade [12], and approximated based on the laryngeal grade on the C-MAC^®^ monitor. A good laryngoscopy view was defined as Cormack-Lehane Grade IIB or better, for ease of data analysis. Finally, for the exploratory difficult intubation assessment, only key variables (Successful intubation, Successful first-pass intubation, Time to intubation) were recorded.

Participant-specific variables were collected as potential covariates, and included age and gender. As not all participants may have had the chance to intubate a real patient before the summative assessment, real-world intubation experience at the summative assessment was included in the analysis as a potential covariate.

### Pilot study

A pilot study was conducted with 20 anesthesia consultants and 20 medical students to determine the feasibility of the study methodology, as well as to validate the intubation technique scoring system that could differentiate between experts and novices. These students were not included in the main study.

### Sample size

In a previous study by our institution [13], the intubation retention success rate at 3 months was reported as 64.5%. Based on preliminary data from the pilot study, the main study was powered to demonstrate a 50% improvement in intubation success rate (alpha = 0.05, beta = 0.10). To demonstrate this, a minimum sample size of 58 participants (29 per group) was needed [14].

### Statistical methods

Trial data was analyzed using SPSS 23.0 for Windows (IBM, Armonk NY, USA). Descriptive statistics were studied for the study population, and stratified according to intervention and control groups (GoPro led vs Non-GoPro led). Between-group statistics were computed and compared using Chi-square test for categorical variables and Student’s t-test for continuous variables.

Primary and secondary outcomes at the summative assessment were analyzed using logistic regression for categorical outcomes, and linear regression with dummy variables for continuous outcomes. Pre-intervention values collected during the formative assessment was accounted for by their inclusion as a covariate during the multivariate analysis. Exploratory variables were analyzed together with primary and secondary outcomes and presented together. A 95% confidence interval was used in analysis. The level of significance for each test was set at α = 0.05, 2 tailed.

A thematic approach was used to analyze qualitative feedback data, with a focus on identifying subjective experiences that could enhance our understanding and interpretation of our quantitative observations.

## Results

**Fig 1:**
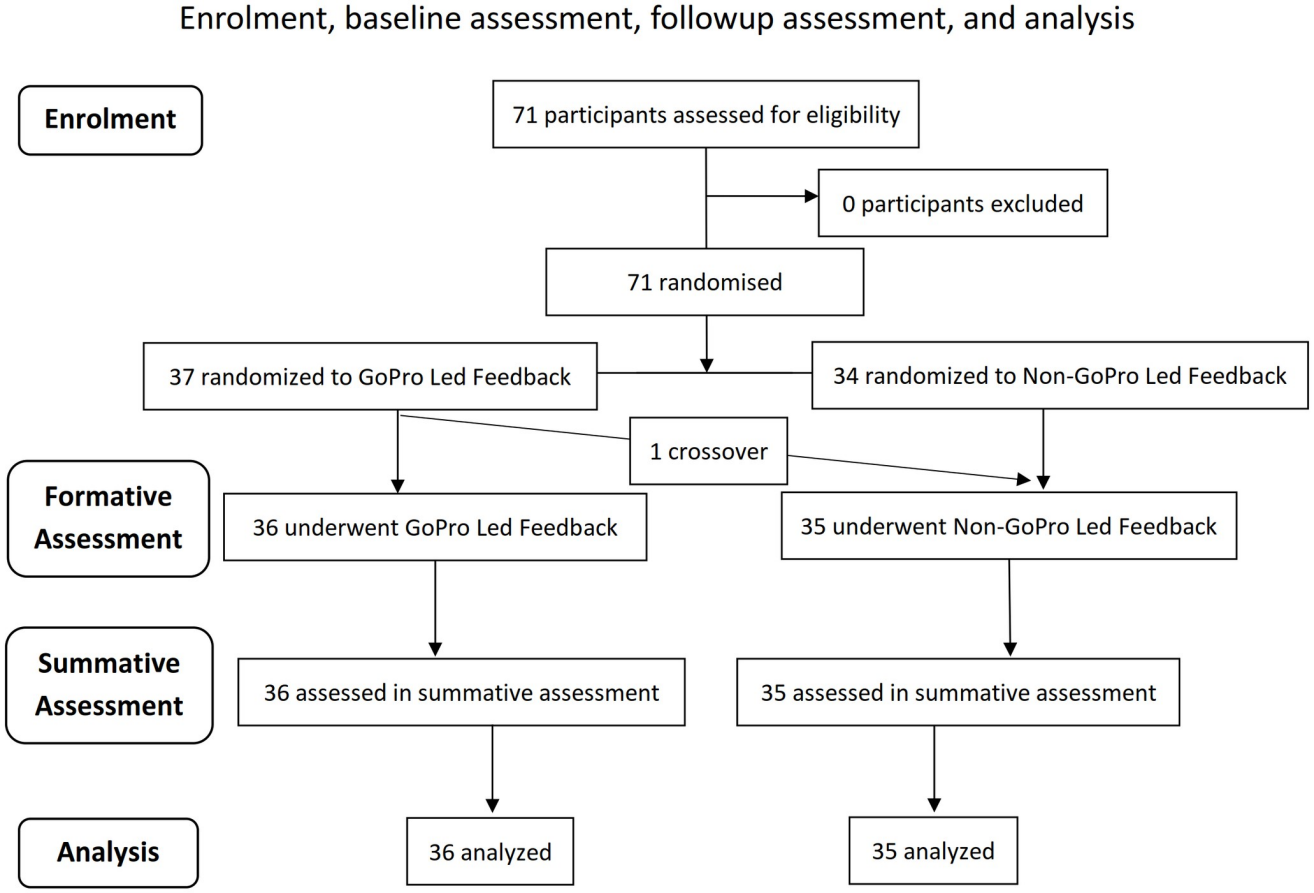
CONSORT diagram of patient enrollment, formative assessment, summative assessment, and data analysis.

**Fig 2:**
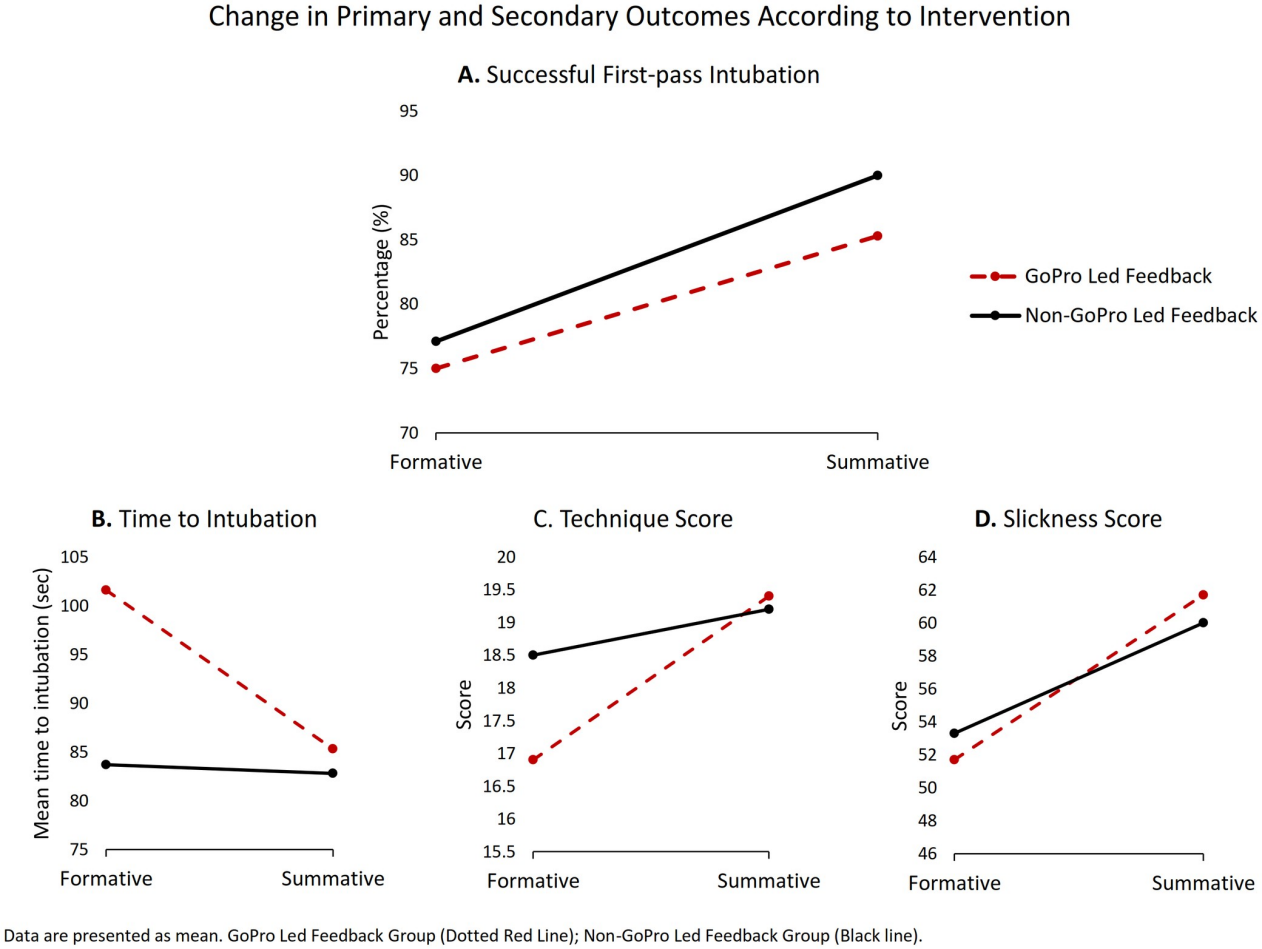
Change in primary and secondary outcomes according to intervention. (A) Primary outcome: Successful first-pass intubation. (B) Secondary outcome: Time to intubation. (C) Secondary outcome: Technique score. (D) Secondary outcome: Slickness score. GoPro Led Feedback Group (Dotted Red Line); Non-GoPro Led Feedback Group (Black Line).

A total of 71 participants were recruited, of which none were excluded (Fig 1). 37 participants were randomized to the GoPro Led Feedback group, and 34 participants were randomized to Non-GoPro Led Feedback group. Due to technical failure of the GoPro camera (from a full SD card) during one of the formative assessments, one participant in the GoPro Led Feedback group did not receive GoPro led feedback. As this crossover was deemed to be completely at random, and no other crossovers occurred, analysis was done in a per-protocol manner, with 36 participants in the GoPro Led Feedback group, and 35 participants in the Non-GoPro Led Feedback group. No participants were lost to follow-up at 3 months. Baseline characteristics during the formative assessment were largely similar between groups (Table 1). Univariate analysis and baseline-adjusted analysis for the summative assessment are presented in Tables 2 and 3. Changes in primary and secondary outcomes between both assessments are also graphed in Fig 2.

**Table 1:** Descriptives and baseline characteristics.

Variable	GoPro Led Feedback Group (n = 36)	Non-GoPro Led Feedback Group (n = 35)	p-value
Gender, male	18 (50.0%)	14 (40.0%)	0.397
Age	22.1 (±0.58)	21.9 (±0.70)	0.359
Intubation experience at summative assessment	24/36 (66.7%)	21 (60.0%)	0.560
**Successful first-pass intubation at baseline**	27/36 (75.0%)	27/35 (77.1%)	0.832
Good laryngeal grade^a^	31/32 (96.9%)	26/30 (86.7%)	0.140
**Time to Intubation (sec) at baseline**	101.6 (±77.1)	83.7 (±43.5)	0.235
Laryngoscopy time (sec)	63.2 (±61.5)	48.6 (±31.3)	0.213
Tube placement time (sec)	38.4 (±37.9)	35.1 (±30.1)	0.692
**Technique score (24-point) at baseline**	16.9 (±3.64)	18.5 (±2.84)	0.056
Preparation score (8-point)	5.64 (±1.89)	6.11 (±1.28)	0.219
Placement score (9-point)	6.36 (±1.69)	6.80 (±1.68)	0.276
Post-intubation score (6-point)	4.72 (±1.47)	5.20 (±0.93)	0.107
Awareness score (1-point)	8/36 (22.2%)	12/35 (34.3%)	0.259
**Slickness score (100-point) at baseline**	51.7 (±14.3)	53.3 (±16.7)	0.656

Data presented as mean (±SD) for continuous data and no. / total (percentage) for categorical data. Primary and secondary outcomes in **bold**.

^a^Reduced sample size due to missing data from technical fault during C-MAC video recording.

**Table 2:** Categorical outcomes at summative assessment with univariate and baseline-adjusted analysis.

Variable	Go-Pro Led Feedback Group at summative assessment	Non-GoPro Led Feedback Group at summative assessment	Univariate p-value	Odds ratio (OR)	95% C.I. for OR		Baseline- adjusted p-value
					Lower	Upper	
**Successful first-pass intubation**^b^	29/34 (85.3%)	27/30 (90.0%)	0.572	0.644	0.140	2.959	0.561
Good laryngeal grade^a^	23/32 (71.9%)	22/29 (75.9%)	0.724	0.813	0.258	2.562	0.838
Awareness score (1-point)	14/33 (42.4%)	10/29 (34.5%)	0.522	1.400	0.499	3.925	0.644
Difficult intubation: Successful difficult intubation	11/36 (30.6%)	15/35 (42.9%)	0.284	0.587	0.221	0.556	
Difficult intubation: Successful difficult first-pass intubation	2/36 (5.6%)	5/35 (14.3%)	0.233	0.353	0.064	1.955	

Data presented as no. / total (percentage). Primary and secondary outcomes in **bold**.

^a^Reduced sample size due to missing data from technical fault during C-MAC video recording.

^b^Reduced sample size due to missing data form technical fault during vantage camera video recording.

**Table 3:** Continuous outcomes at summative assessment with univariate and baseline-adjusted analysis.

Variable	GoPro Led Feedback Group at summative assessment	Non-GoPro Led Feedback Group at summative assessment	Univariate p-value	Regression coefficient (B)	95% C.I. for B		Baseline-adjusted p-value
					Lower	Upper	
**Time to Intubation (sec)**	85.3 (±70.4)	82.8 (±55.9)	0.874	2.557	-29.506	34.620	0.969
Laryngoscopy time (sec)	39.6 (±22.8)	33.8 (±17.8)	0.272	5.725	-4.606	16.057	0.418
Tube placement time (sec)	45.8 (±60.6)	48.9 (±46.8)	0.817	-3.169	-30.489	24.152	0.849
*Number of subjects analyzed*^a^	*(n = 34)*	*(n = 30)*					
**Technique score (24-point)**	19.4 (±4.13)	19.2 (±2.98)	0.837	0.191	-1.661	2.043	0.858
Preparation score (8-point)	6.52 (±1.42)	6.62 (±1.32)	0.764	-0.106	-0.804	0.593	0.884
Placement score (9-point)	7.30 (±1.85)	6.79 (±1.63)	0.257	0.510	-0.381	1.401	0.299
Post-intubation score (6-point)	5.12 (±1.50)	5.41 (±1.18)	0.400	-0.293	-0.984	0.399	0.366
**Slickness score (100-point)**	61.7 (±14.2)	60.0 (±14.1)	0.645	1.667	-5.529	8.862	0.631
*Number of subjects analyzed*^a^	*(n = 33)*	*(n = 29)*					
Difficult intubation: Time to Intubation (sec)	235.8 (±145.6)	141.0 (±86.7)	0.052	94.800	-1.005	190.605	
*Number of subjects analyzed*^a^^b^	*(n = 10)*	*(n = 15)*					

Data presented as mean (±SD). Primary and secondary outcomes in **bold**.

^a^Reduced sample size due to missing data form technical fault during vantage camera video recording.

^b^Analysis only includes participants who successfully intubated difficult manikin

All participants were able to successfully intubate in both the formative and summative assessment. Both study groups had a higher percentage of successful first-pass intubation at the summative assessment as compared to the formative assessment. No statistically significant differences were found between the GoPro group and the non-GoPro group at summative assessment (85.3% vs 90.0%, p = 0.572).

The GoPro group tended to need more time to intubate at baseline (101.6 seconds vs 83.7 seconds, p = 0.235), however they improved after the formative assessment, such that both groups converged at the summative assessment (85.3 seconds vs 82.8 seconds, p = 0.874). On baseline-adjusted analysis, however, the GoPro group did not significantly improve more than the non-GoPro group.

Both groups had a similar intubation technique score on average at their summative assessment (19.4 points vs 19.2 points, p = 0.837). Both groups also performed similarly in terms of their intubation slickness score during the summative assessment (61.7 vs 60.0 points, p = 0.645).

Compared to the Laerdal^®^ Airway Management Trainer, most participants were not able to intubate the Difficult Airway Management Simulator. No statistically significant differences were found between the GoPro group and the non-GoPro group in terms of all 3 exploratory outcomes.

### Participant survey

**Fig 3:**
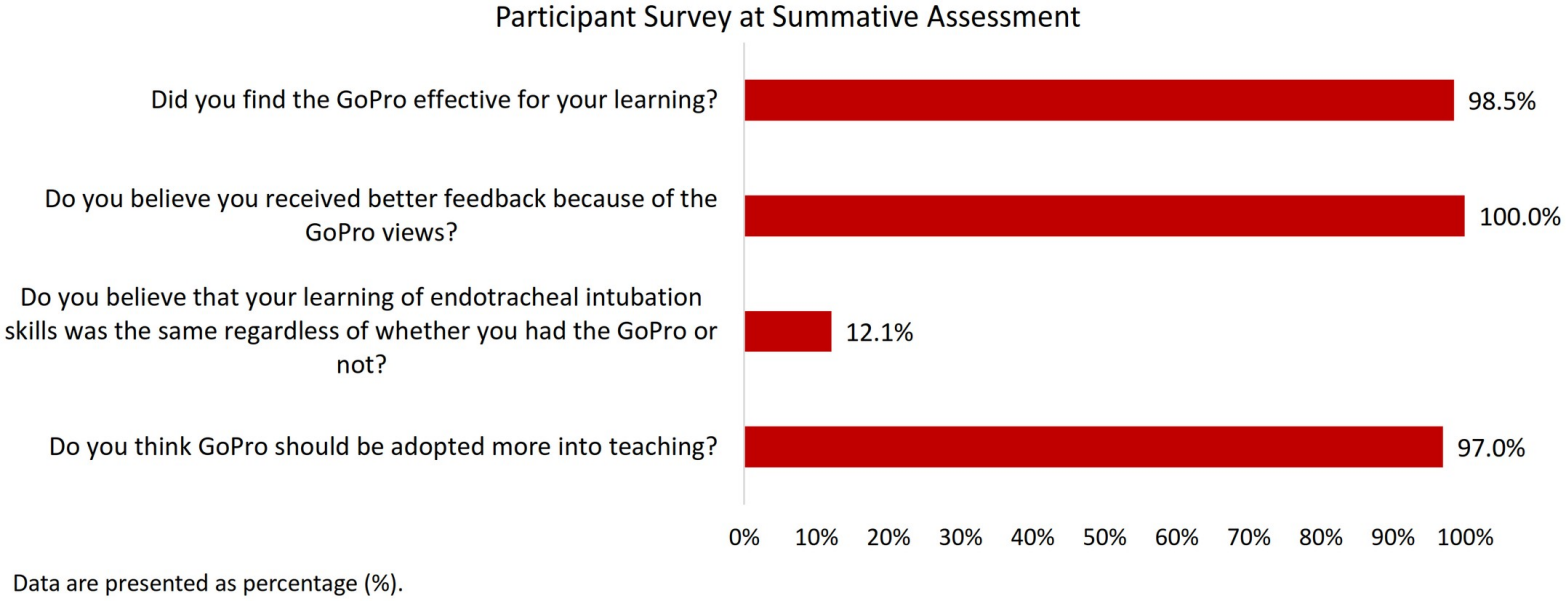
Participant survey at summative assessment.

Our survey results are presented in Fig 3. The overwhelming majority of participants found the GoPro effective for their learning (98.5%), felt that they received better feedback because of the GoPro (100%), and felt that the GoPro should be adopted more into teaching (97%). Few (12.1%) believed that their learning would be the same regardless of whether they had the GoPro or not.

### Qualitative analysis

The majority of participants comments were positive in nature.

The predominant theme was the ability for an improved assessment using GoPro video. Many participants felt that the GoPro was able to improve their feedback session in a specific way. This ranged from being able to *review mistakes* and *assess intubation competency using the GoPro after the intubation*, to providing an *objective and accurate documentation* of the participant’s performance. One participant felt that the GoPro enabled a *step-by-step review* of his technique, while another felt that it gave *the ability to pause*, *rewind*, *and focus on important learning points*. Participants also felt that the GoPro helped them to have a *better perspective on what their strengths and weaknesses were*, as well as their *blind spots*.

Another common theme was how *small details* that would otherwise be missed were brought to light. These participants found details or mistakes they *made way often in the past but never noticed*, *did not realize initially*, or *might not be aware of while doing the intubation*. As illustrated in one participant’s experience, *“The tutor was able to point out my mistakes*, *even the minor ones after reviewing the video*, *which could not be done without the GoPro”*.

Many participants welcomed the benefit of having visual footage of their attempt, and having a viewpoint from their point of view led to improved understanding. Participants valued how they were able to *see the view they were seeing*, *see what went wrong or right from their point of view*, *see things from a different angle*, or *understand mistakes that they are usually not able to ‘visualize’*. Particularly insightful was a feedback that it *made more sense to see a pair of hands from his point of view doing the intubation*.

Interestingly, for many participants there was a sense that there were learning points that could be accrued even without the presence of a tutor. These participants appreciated the opportunity for *self-evaluation and awareness*, and becoming *more conscious of their mistakes*. They also felt that just *re-watching the intubation attempt reinforces correct technique*. One participant mused that *it would have been great if he had access to his GoPro videos for revision purposes*. Some appreciated the usefulness of the simulation as an *additional platform* for intubation training.

## Discussion

Overall, when surveyed, the overwhelming majority of participants believed that the addition of a GoPro was helpful for both feedback and learning. This was seen across all survey questions. As with the survey results, our qualitative thematic analysis showed almost all participants reported positive experiences with GoPro led feedback. Common themes in qualitative analysis were: the ability for an improved assessment, greater identification of small details that would otherwise be missed, and usefulness of the point-of-view footage in improving understanding. The positive reactions from our participants demonstrate how the GoPro’s unique vantage point and ability to record the intubation attempt may assist in intubation teaching, maximizing the yield from simulation training.

However, we were unable to show a statistically significant difference between the GoPro Led Feedback group and the Non-GoPro Led Feedback group in terms of first pass intubation success, time to intubation, intubation technique score, and intubation slickness score at the summative assessment. When the formative assessment baseline was taken into account, the GoPro Led Feedback group did show a greater improvement in the time to intubation and the intubation technique score, however these differences were not statistically significant.

There are several possible reasons why a quantitative difference in results were not observed. One possibility is that the GoPro Led Feedback session was ineffective, however the positive feedback from our participants suggests otherwise. More likely, as the study intervention consisted of a single feedback session, participants may have had insufficient practice to achieve a significant improvement in intubation proficiency. It is well known that intubation is a skill that requires repeated practice to establish competency [11, 15], and Konrad et al estimated that an average of 57 intubations is required for a 90% intubation success rate [16].

While organising many more feedback sessions will be ideal, the logistics of carrying out many more sessions may be challenging. Based on participant feedback as well as tutor experiences, we suggest providing participants with additional time with the manikin and the GoPro for self-directed and peer review learning. This would not take up precious tutor time, and would provide participants with a longer practice period. Additionally, GoPro videos may also be disseminated to participants so that they can access their previous intubation videos according to their own schedule. This allows them to better revise and understand their mistakes, and internalize their successful intubations. These adjunctive improvements to the feedback session could further improve their learning value, and reduce the number of practice sessions needed to achieve competency.

There are some limitations to this study. The study was powered to look at a 50% improvement in intubation success rate, and we were unable to show statistically significant differences in the outcomes measured. Larger studies will be needed to confirm our results. Additionally, the focus of the study was on manikin-based intubations, and studies in real-world intubations will be useful in expanding the GoPro’s role to intubation teaching beyond the simulated setting.

In conclusion, the GoPro’s unique vantage point and ability to record the intubation attempt allow for improved assessment, greater identification of small details, and improved understanding in intubation teaching and feedback. While it is a promising tool to improve simulation-based intubation teaching, an appreciation of its merits is needed to design a simulation session that maximizes its effectiveness. These should be taken into account in future trials, and can also be applied across the field to other point-of-view camera led teachings.

## Supporting information

S1 Appendix Intubation “technique score” checklist used to assess intubation technique. (DOCX)
