# Supplementary material for: Use of GoPro point-of-view camera in intubation simulation—A randomized controlled trial
Source: PLoS One. 2020 Dec 1;15(12):e0243217. doi: 10.1371/journal.pone.0243217 (PMC7707475; doi:10.1371/journal.pone.0243217)
Supplement: S1 Appendix — (DOCX) [file pone.0243217.s001.docx]

**Supporting information**

**S1 Appendix:** Intubation “technique score” checklist used to assess intubation technique.

| Preparation score (8 points) | 1) Wear gloves |
| --- | --- |
|  | 2) Check that laryngoscope light bulb is working |
|  | 3) Check that syringe is available |
|  | 4) Test cuff to exclude air leak |
|  | 5) Check that suction device is working |
|  | 6) Assemble bag-valve-mask device correctly |
|  | 7) Connect mask to oxygen source |
|  | 8) Preoxygenate patient with mask |
| Intubation score (9 points) | 1) Correctly perform head-tilt chin-lift maneuver |
|  | 2) Adequately open mouth before laryngoscope insertion |
|  | 3) Hold laryngoscope in left hand |
|  | 4) Insert laryngoscope into right side of mouth |
|  | 5) Sweep tongue to the left with laryngoscope blade |
|  | 6) Advance laryngoscope into vallecula |
|  | 7) Traction of laryngoscope handle along correct axis without levering on teeth |
|  | 8) Visualise vocal cords |
|  | 9) Correctly guide endotracheal tube into mouth and through cords |
| Post-intubation score (6 points) | 1) Inflate cuff |
|  | 2) Connect bag-valve-mask device |
|  | 3) Check for chest rise |
|  | 4) Perform 3-point auscultation |
|  | 5) Check for ETCO2 |
|  | 6) Securely tape endotracheal tube in place |
| Awareness score (1 point) | 1) Show awareness of oxygen saturation |
